# Anatomical Findings of Renal and Urological Abnormalities in Cardiac Catheterization of Children with congenital heart diseases – A Single Center Experience

**DOI:** 10.5339/qmj.2021.54

**Published:** 2021-10-19

**Authors:** Mohammad Reza Khalilian, Abbas Mollatayefeh, Tahmineh Tahouri, Arash Mahdavi, Reza Dalirani

**Affiliations:** ^1^Department of Pediatrics, School of Medicine, Shahid Modarres cardiovascular Medical and Research Center, Shahid Beheshti University of Medical Sciences, Tehran, Iran.; ^2^Department of Pediatrics, School of Medicine, Mofid Children's Hospital, Shahid Beheshti University of Medical Sciences, Tehran, Iran.; ^3^Department of Radiology, School of Medicine, Shahid Modarres Hospital, Shahid Beheshti, University of Medical Sciences, Tehran, Iran.; ^4^Department of Pediatrics, School of Medicine, Pediatric Nephrology Research Center, Mofid Children's Hospital, Shahid Beheshti University of Medical Sciences, Tehran, Iran.

**Keywords:** congenital heart disease, children, anatomical findings, angiography

## Abstract

ackground and aims: Congenital heart disease (CHD) is described as an abnormality in the heart structure or intra-thoracic great vessels that leads to functional problems. Since most of these disorders require medical and surgical interventions identifying concomitant disorders such as renal and urinary tract abnormalities is of great importance in the management of these patients. The present study aimed to investigate the relative frequency of abnormal kidney and urinary tract findings in abdominal cineangiography during cardiac catheterization of patients with CHD in Shahid Modarres Cardiovascular Medical and Research Center.

Methods: The present study was performed prospectively on 545 patients aged < 18 years with CHD who underwent cardiac catheterization and concurrent abdominal cineangiography in Shahid Modarres Cardiovascular, Medical and Research Center, Tehran, Iran during a three-year period. The required data were extracted using a researcher-made questionnaire from patients’ electronic medical files.

Results: Of a total of 545 patients in this study, 26 had both CHD and renal or urinary tract malformation. Patent ductus arteriosus was the most common CHD in patients with renal or urinary tract malformations (odds ratio: 1.2, 95%, CI: 2.25–11.63). In this study, the most common renal and urinary malformations among CHD patients was partial duplication of the kidney followed by Ureteropelvic Junction Obstruction.Conclusion: Since the prevalence of renal and urinary tract malformations is higher in CHD patients, performance of concurrent abdominal cineangiography during cardiac catheterization may lead to early diagnosis and treatment as well as better pre- and post-operative management of patients.

## Introduction

Congenital heart disease (CHD) is described as an abnormality in the heart structure or the intra-thoracic great vessels that leads to functional problems.^1^ Generally, 22% to 45% of CHD patients have concomitant extra-cardiac anomalies or genetic syndromes.^2^ The prevalence of renal and urinary tract abnormalities in CHD is estimated at approximately 7.5%–12.5%, which is higher than that in the general population.^3^ These renal and urinary tract abnormalities include renal agenesis, cystic kidney disease, pelvicaliectasis, hydronephrosis, ectopic kidneys, horseshoe kidneys, and duplex kidneys. Interestingly, there is no association between the type of cardiac lesion and renal or urinary tract anomalies.^4^

Current studies demonstrate a genetic correlation between CHD and renal or urinary tract anomalies, namely mutations in cilia genes may be associated with both CHD and kidney or urinary tract anomalies.^5^ On the other hand, in some CHD patients, in particular cyanotic patients, some degree of kidney dysfunction, such as a diminished glomerular filtration rate, azotemia, nephrotic syndrome and proteinuria, is possible. The prevalence of these functional abnormalities is directly related to the severity of cyanosis and its associated polycythemia.^6^

Since the renal abnormalities mentioned above can be asymptomatic and therefore not be detected by parents, routine assessment of the urinary system in CHD patients seems reasonable.^4^

Herein, we investigated random anatomical findings of renal and urological abnormalities in cardiac catheterization of patients with congenital heart diseases in Shahid Modarres Cardiovascular, Medical and Research Center of Tehran, Iran. The aim of this study was to evaluate the prevalence and type of renal or urinary tract malformations in CHD patients who have no signs or symptoms of kidney or urologic disease, which is valuable in the management of these patients, mainly in the post-operative period.

## Subjects And Methods

In this prospective cross-sectional study, 545 patients aged < 18 years with congenital heart disease who underwent cardiac catheterization and concurrent abdominal cineradiography in Shahid Modarres Cardiovascular, Medical and Research Center, Tehran, Iran, between April 2016 and December 2019 were enrolled. The ethics committee of Shahid Beheshti University of Medical Science approved the study protocol by reference number of IR.SBMU.MSP.REC.1398.828.

Patients, who enrolled in the study, had no previous history of kidney or urinary problems and their laboratory tests including blood urea nitrogen (BUN), creatinine, urinary analysis, and culture, prior to catheterization, were all normal. Abdominal cineangiography was performed after contrast injection during cardiac catheterization. Demographic and clinical data including age, gender, type of congenital heart disease and renal abnormality collected using a researcher-made questionnaire from patients’ electronic medical records. The exclusion criteria were the presence of any other extra-cardiac malformation or genetic syndrome and the unavailability of patient's medical records. All abdominal cineangiography results were reviewed and reported by an experienced radiologist.

### Statistical analysis

Statistical analysis was performed utilizing SPSS Statistics version 23 for Windows (IBM Corp.; Armonk, NY, USA) on two descriptive and inferential levels. For descriptive statistics, statistical indices of graphs, frequency tables, relative frequency distribution, cumulative frequency, mean percentage, and standard deviation were used. For inferential statistics, logistic regression analysis was used, which examines the effect of two variables on a two-state variable simultaneously. P values of 0.05 or less were considered statistically significant.

## Results

The median age of the patients was 2 years (interquartile range (IQR): 0.9–6). Of these patients, 140 were < 1 year old, 361 patients were 1–12 years old, and 44 patients were >12 years old.

Of all 545 patients, 296 (54.3%) were males, and 249 (45.7%) were females. Thirteen 13 (4.4%) male patients and 13 (3.5%) female patients had renal or urinary tract malformations. No differences with respect to gender were noted between groups (p = 0.582).

Of the total study population, 380 patients were acyanotic, and 165 patients were cyanotic. In the acyanotic group, 17 patients (4.5%) and in the cyanotic group 9 patients (5.5%) had renal or urinary tract malformations. Logistic regression analysis showed no significant relationship between renal or urinary tract malformation and the patients’ oxygen saturation status (p = 0.601).

In this study, the total prevalence of renal malformations in CHD patients was 4.7%. The most frequent renal and urinary tract malformation was duplication of the kidney (partial or complete), which was seen in 5 of 26 CHD patients with renal or urinary tract malformations. Table 1 shows the frequency of each renal or urinary tract malformation in CHD patients.

Of a total of 26 patients with renal or urinary tract malformation, the most common CHD was patent ductus arteriosus (PDA), with a frequency of 42.3%. Logistic regression analysis showed that the frequency of PDA was significantly higher in patients with renal or urinary tract malformation (Odds ratio: 1.2, 95% CI: 2.25–11.63). Table 2 shows the frequency of each CHD in patients with renal and urinary tract malformation.

## Discussion

In this study, we examined the random anatomical findings of renal and urological abnormalities in abdominal cineangiography of patients with CHDs during their cardiac catheterization.

Abnormalities in the size and function of the kidneys in patients with CHD have shown by previous studies. According to Scholes et al., the kidneys of newborns with CHD are larger than normal. Neonates with cyanotic CHD may have large or normal sized kidneys, whereas in patients with left heart obstruction, larger than normal kidneys are common.^7^

A review of the literature revealed that the overall prevalence of kidney malformations is approximately 0.3%–0.6% in the general population, but in CHD patients, it is variable. Hamda et al. evaluated ultrasonic kidney findings in CHD patients and found that approximately 94% of patients have normal kidneys.^8^ This is statistically identical to our study in which the prevalence of renal and urinary malformations in CHD patients was 4.7%. Similarly, it was also seen in the study of Niedenbach et al. that approximately 95% of CHD patients did not have concomitant kidney disease.^9^

The results of this study revealed that, similarly to other studies in this field, there is no significant relation between the gender of CHD patients and the presence of kidney or urinary tract malformation (P = 0.582). Furthermore, the prevalence of urological abnormalities in cyanotic and acyanotic patients was not significantly different. Based on a study by Dittrich et al., the incidence of nephropathy in cyanotic CHD is increased mainly due to glomerular injury and is directly related to the duration of cyanosis and level of hematocrit.^10^ Mohamed et al. also found that patients with cyanotic CHD were at increased risk of both tubular and glomerular dysfunction in comparison to acyanotic CHD patients.^11^ Nevertheless, the current study showed no significant relationship between renal abnormalities and patient's oxygen saturation status (P = 0.582).

In our study, of the 26 patients with renal or urinary tract malformations, the most common CHD was PDA. However, a study by Jiang et al. showed no significant relationship between renal malformation variants and CHD types.^3^ Similarly to our study, Barakat, et al. showed that among all CHDs, PDA has the most association with kidney malformations followed by VSD^12^. Conversely, in a study by Jiang et al. the most common CHD in patients with renal and urinary tract anomalies was ASD; this discrepancy may be because they used ultrasound as a screening tool for evaluation of kidney and urinary tract malformations, whereas we examined the prevalence of these abnormalities only in patients who underwent cardiac catheterization which are usually have more complex CHDs.

Since the patients in our study, had no signs or symptoms of urological or renal problems, assessment of their urinary system during catheterization can reveal anomalies which could not be detected by parents or even a physician. Though, most congenital anomalies of kidney and urinary tract do not require surgery and have no effect on the outcomes of congenital heart surgery; referring to a pediatric nephrologist before and after the CHD surgery seems reasonable.^3^ The risk of urinary tract infection in the post operation period of the patients with kidney or urinary tract anomalies may be increased. This increase in urinary tract infection rate may be related to the cross-clamp time, bypass time, and the complexity of the heart surgery. Therefore, the type and the duration of post-operative prophylactic antibiotics in these patients may be different from the others. Besides, acute kidney injury following the cardiac surgery and the cardiopulmonary bypass is possible in these patients. Optimizing hemodynamic status before, during, and after the surgery may help prevent such acute kidney injury.^8^

The principal limitation in our study was its single center design. In this regard, Multi-center studies with large sample size are required. Another limitation was that only patients who underwent catheterization for angiographic intervention or preoperative assessment were included in this study. Therefore, patients for whom other imaging modalities such as CT angiography or cardiac MRI were performed instead of catheterization were not included in the study, which could affect the results of the present study.

In summary, the results of our study proved a higher prevalence of renal or urinary tract malformations in CHD patients than in the general population, which agrees with previous studies. This highlights the role of evaluation of the urinary system in management of CHD patients, mainly in the post-operative period.

## Conclusion

The higher prevalence of renal and urinary tract malformations in patients with CHD highlights the role of concurrent abdominal cineangiography during cardiac catheterization for better pre- and post-operative management of patients. PDA is the most common CHDs in patients with renal or urinary tract malformations.

## Acknowledgment

The authors are grateful to all parents and patients for their participation in this study.

## Conflict Of Interest

The authors declare that they have no conflict of interest.

### Key message

Most congenital heart diseases require identification of concomitant disorders, such as those of the renal and urinary tract, because of their great importance in the management of these patients.

## Figures and Tables

**Table 1:** Types of renal and urinary tract malformations in CHD patients

Types of renal malformations	Number

Partial duplication of kidney	5

Unilateral malrotation of kidney	4

UPJO^a^	4

Ext-renal pelvis	3

Kidney agenesis	2

Circumcaval ureter	2

Complete duplication of kidney	1

Medial deviation of left kidney	1

Narrowing of left ureter	1

Spiral appearance of bilateral ureters	1

Beading of bilateral ureters	1

Ptotic kidneys	1

Total	26


a: Ureteropelvic Junction Obstruction

**Table 2:** Frequency of each CHD in patients with renal and urinary tract malformations

Type of CHD	number	frequency

PDA^a^	11	42.5%

DORV^b^	4	15.6%

TOF^c^	2	7.6%

ASD^d^	1	3.8%

VSD^e^	1	3.8%

AVSD^f^	1	3.8%

Liver AVM^g^	1	3.8%

P.A^h^	1	3.8%

PS^i^	1	3.8%

T.A^j^	1	3.8%

TGA^k^	1	3.8%

Shone complex	1	3.8%

total	26	100%


a: Patent Ductus Arteriosus, b: Double Outlet Right Ventricle, c: Tetralogy of Fallot, d: Atrial Septal Defect, e: Ventricular Septal Defect, f: Atrioventricular Septal Defect, g: Arteriovenous Malformation, h: Pulmonary Atresia, i: Pulmonary Stenosis, j: Tricuspid Atresia, k: Transposition of the Great Arteries
